# Isolated slow orthostatic tremor of the trunk

**DOI:** 10.1186/s42466-022-00216-6

**Published:** 2022-10-17

**Authors:** N. Ahmad Aziz, Marcus Grobe-Einsler, Oliver Kaut

**Affiliations:** 1 Department of Neurology, University Hospital Bonn, Venusberg-Campus 1, 53127 Bonn, Germany; 2 German Center for Neurodegenerative Diseases (DZNE), Bonn, Germany

**Keywords:** Slow orthostatic tremor, Essential tremor, Trunk tremor, Propranolol, Primidon

## Abstract

Slow orthostatic tremor is an extremely rare movement disorder with relatively low-frequency tremor (< 13 Hz) in the legs and trunk, which is evoked by standing. There is still much controversy regarding its precise etiology. Here we present a 57 year-old female patient with a slow orthostatic tremor variant who experienced progressive gait disturbances since six years due to *isolated* trunk tremor. Potential symptomatic causes of tremor and other neurological co-morbidities were excluded through an exenstive clinical, laboratoy and imaging work-up. Subsequently, a combined treatment with propranolol and primidone was started, which resulted in almost complete resolution of the trunk tremor. Given that the slow trunk tremor in this patient almost completely resolved after therapy with a low-dose propranolol and primidone, considered first line drugs for the treatment of essential tremor, this case illustrates that isolated orthostatic trunk tremor may occur as a rare variant of essential tremor.

**Supplementary Information:**

The online version contains supplementary material available at 10.1186/s42466-022-00216-6.

## Case report

Slow orthostatic tremor is defined as a relatively low-frequency tremor (< 13 Hz) in the legs and trunk, which is evoked by standing. It is an extremely rare movement disorder with only 82 cases reported to date [1, 2]. There is still much controversy regarding its precise etiology, with some sources claiming it to be a variant of either the classical orthostatic tremor, or other tremor disorders given frequent coexistent neurological co-morbidity, most notably Parkinson’s disease and other parkinsonian syndromes as well as essential tremor [1, 3, 4].

**Fig. 1:**
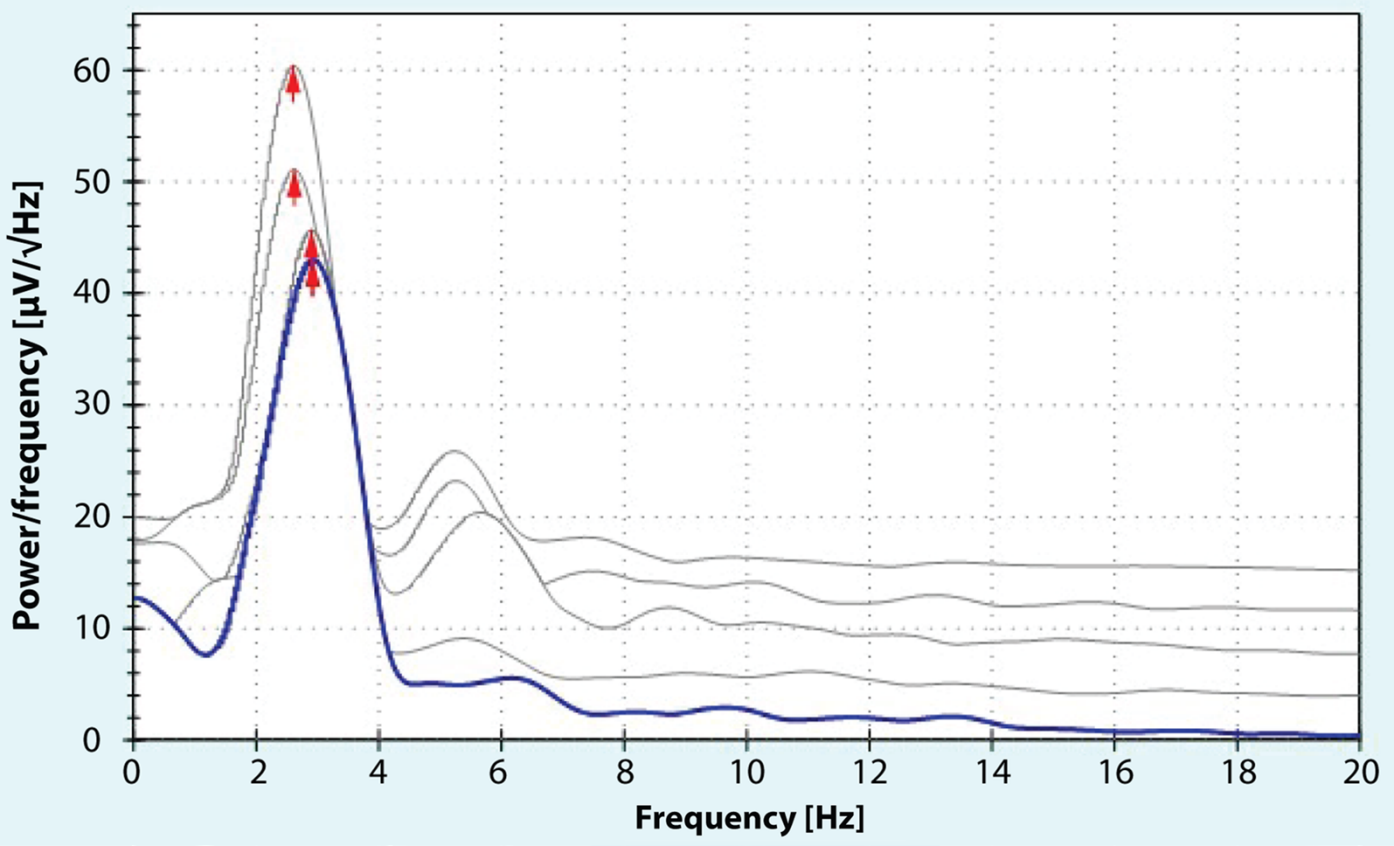
Tremor analysis using surface accelerometers attached to the paravertebral muscles (at thoracic level 12 on the right) revealed high-amplitude rhythmic oscillations with a peak frequency of 2–4 Hz

Here we present a 57 year-old female patient with a slow orthostatic tremor variant who experienced progressive gait disturbances since six years due to *isolated* trunk tremor. The trunk tremor was evoked by both standing and walking (Additional file 1: Video). Apart from a surgically treated cervical disc herniation, type II diabetes, arterial hypertension and hypercholesterolemia, her medical history was unremarkable. There was no alcohol response or a family history of tremor or other neurological diseases. Exposure to neurotoxic compounds was ruled out. Similarly, except for isolated orthostatic trunk tremor, the neurological examination was also unremarkable; in particular, there were no (subtle) signs of parkinsonism or dystonia present during repeated neurological examinations. The deep tendon reflexes as well as extensive nerve conduction studies were normal, except indications for an (old) lesion of the somatosensory fibers to the right leg based on an absent cortical somatosensory evoked potential of the right tibial nerve. However, there were no indications for motor fiber lesions on either side, suggesting an isolated focal sensory deficit. Accelerometry of the rectus abdominis and paravertebral muscles revealed a rhythmic tremor with a frequency of 2–4 Hz (Fig. 1). Needle electromyography of the paravertebral muscles at the level of the 12th thoracic vertebra confirmed rhythmic bursts with a peak frequency of 2.5 Hz. The symptoms had started six years before initial presentation following a period of putative vertebrobasilar ischemia. However, brain as well whole spine MR imaging was normal. Similarly, extensive laboratory examinations, including complete blood count, electrolytes, kidney, liver and thyroid function tests, as well as vitamin levels were all within the normal range. Cerebrospinal fluid examination did not reveal any signs of (chronic) infection or the presence of anti-neuronal antibodies; the panel used, assessed in both serum and cerebrospinal fluid, included the following target antigens: Amphiphysin, CV2, PNMA2 Ma-2/Ta, Ri, Yo, Hu, Recoverin, SOX1, Titin, GAD 65, Zic, Tr (DNER), NMDAR, AMPA1R, AMPA2R and CASPR2.

Initial treatment with perampanel, which has been reported to be effective against primary orthostatic tremor in recent case series [5, 6], did not noticeably affect the symptoms. However, subsequent treatment with propranolol (80 mg sustained-release once daily) led to improvement of her symptoms. Additional uptitration of a low-dose of primidone until 75 mg daily resulted in almost complete resolution of the trunk tremor (Additional file 1: Video). At the last follow-up visit, more than three months after the initiation of propranolol, there was still an excellent persisting treatment response. The dramatic clinical improvement was reflected in the resumption of her professional duties and a substantial improvement of her quality of life.

Propranolol and primidone are considered first line drugs for the treatment of essential tremor, and in combination are more potent than each one separately. Given that the slow trunk tremor in this patient almost completely resolved after therapy with a low-dose propranolol and primidone, this case illustrates that isolated orthostatic trunk tremor may occur as a rare variant of essential tremor. Consequently, at least a trial of medications used for the treatment of essential tremor may be warranted in patients presenting with slow orthostatic tremor. The Consensus Statement on the Classification of Tremors from the Task Force of Tremor of the International Parkinson and Movement Disorder Society proposed the designation “pseudo-orthostatic tremor” for describing all orthostatic tremors with a frequency < 13 Hz. [7] Our case, which would fit this designation, nicely illustrates that the category “pseudo-orthostatic tremor” is likely to encompass a highly heterogeneous group of tremors and their variants as well as corresponding etiologies, necessitating a systematic search for the underlying cause and tailored treatment on individual basis.

## Supplementary Information

**Additional file 1**. The video shows a patient with an extremely rare variant of slow orthostatic tremor with isolated trunk tremor that is elicited by standing and walking. The first part depicts the neurological examination at baseline, whereas the second part shows almost complete resolution of symptoms following treatment. Please note that although the video suggests a reduced arm swing on the right side, this was only a momentary occurrence and thus not a consistent feature of the patient’s symptomatology.

## Data Availability

Not applicable.
